# Intervention mapping for the development of a strategy to implement the insurance medicine guidelines for depression

**DOI:** 10.1186/1471-2458-11-9

**Published:** 2011-01-05

**Authors:** Feico Zwerver, Antonius JM Schellart, Johannes R Anema, Kathelijne C Rammeloo, Allard J van der Beek

**Affiliations:** 1 VU University Medical Center, Department of Public and Occupational Health, EMGO Institute for Health and Care Research, Amsterdam, The Netherlands; 2 Research Center for Insurance Medicine, collaboration between AMC-UWV-VUmc, Amsterdam, The Netherlands; 3 Dutch National Institute for Employee Benefits Schemes, Amsterdam, The Netherlands

## Abstract

**Background:**

This article describes the development of a strategy to implement the insurance medicine guidelines for depression. Use of the guidelines is intended to result in more transparent and uniform assessment of claimants with depressive symptoms.

**Methods:**

The implementation strategy was developed using the Intervention Mapping (IM) method for alignment with insurance-medical practice. The ASE behavioural explanation model (Attitude, Social Influence and Self-Efficacy) was used as theoretical basis for the development work. A literature study of implementation strategies and interviews with insurance physicians were performed to develop instruments for use with the guideline. These instruments were designed to match the needs and the working circumstances of insurance physicians. Performance indicators to measure the quality of the assessment and the adherence to the guidelines were defined with input from insurance physicians.

**Results:**

This study resulted in the development of a training course to teach insurance physicians how to apply the guidelines for depression, using the aforementioned instruments. The efficacy of this training course will be evaluated in a Randomized Controlled Trial.

**Conclusions:**

The use of IM made it possible to develop guideline support instruments tailored to insurance medical practice.

## Background

**Figure 1:**
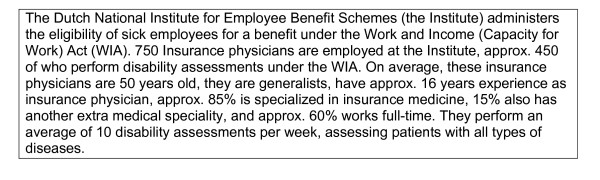
**Insurance physicians in the Netherlands; source: R**. Steenbeek [4].

Depression is an enormous health problem, which is responsible for 11% of disability worldwide [1]. The WHO predicts that by 2020, depression will be second only to heart disease as a cause of lost disability-adjusted life-years and untimely death [2]. Through social insurance, employees can claim compensation when they lose (part of) their income due to disability. To determine these disability benefit claims, disability assessments are carried out by specialized physicians, who have to evaluate the claimants' medical status and functional capacities with regard to vocational rehabilitation [3]. In the Netherlands, these assessments are performed by insurance physicians (IPs) who work for the Dutch Institute for Employee Benefit Schemes (Institute). The context of insurance medicine in the Netherlands is presented in Figure 1[4]. Worldwide, physicians are involved in similar assessments, even though national practices, social systems and, disability legislation, may vary considerably [5].

In the Netherlands, 19 diagnosis specific guidelines, including depression, have recently been developed for use in insurance-medical practice [6,7]. These guidelines are intended to serve as a reference framework that can help IPs to make their disability assessments more evidence-based and more standardized [8,9]. IPs have to know all 19 guidelines and apply them in practice because they are generalists. The guidelines were subsequently implemented top-down by the Institute in the period between 2007 and 2009. This tight schedule of guideline implementation did not leave much time for the IPs to really apply all these guidelines. During this period, two guidelines were sometimes implemented in one single afternoon session. Unfortunately, little attention was paid to the needs of the IPs. Except for lack of time, the implementation of evidence-based guidelines in health care practice has proven to be difficult anyway [10]. Implementing guidelines requires the planning of complex changes in practice. Potential barriers at various levels need to be overcome, such as the nature of the guidelines, the characteristics of the physicians involved, and the social, organizational, economical and political context [11-14]. Using the intervention mapping method (IM), it is possible to make provisions for the needs of the users and those around them, and to draw upon scientific theory and evidence, in the implementation of protocols and guidelines. Since 1998, IM has mainly been used for planning theory- and evidence-based health promotion programs [15-17]. However this method has now reached the field of occupational health medicine, where it is being used to support the development of intervention programs focussing on work disability [18-20]. This article describes the use of intervention mapping for the development of a strategy for the implementation of insurance medicine guidelines for depression. The aim was to answer the following core question: What approach should be taken to implement these guidelines, in order to ensure effective use by insurance physicians? A randomized controlled trial (RCT) will in due course be carried out to compare the efficacy of the implementation strategy described in this article with conventional implementation methods.

## Methods

**Figure 2:**
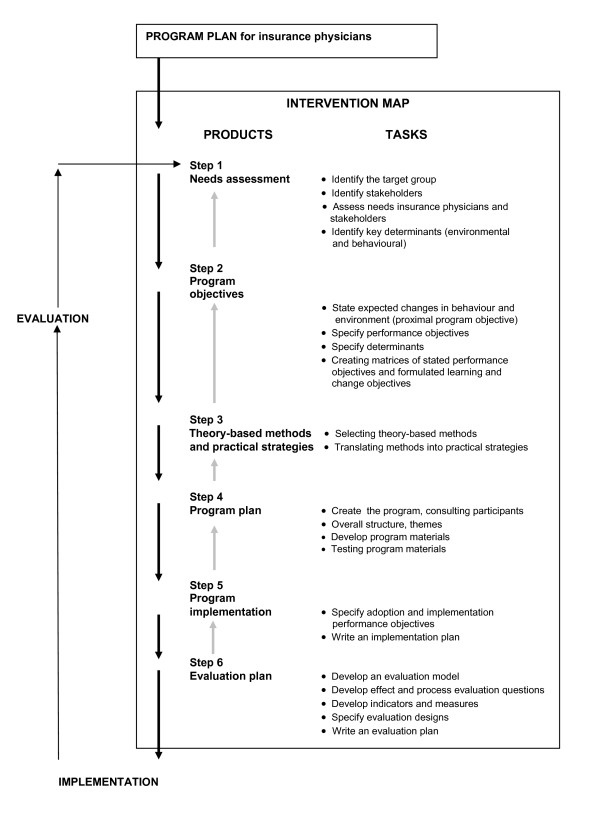
**Intervention Mapping Process, developed by Bartholomew et al **[15]. Note: The term "insurance physician" was added to the original plan.

IM, developed in the nineties by Bartholomew et al. [15,21], is a planning instrument that maps out the development process of an intervention from the basic needs to the potential solution. IM provides a stepwise process for decisions, based on theory and evidence. It consists of six steps, which are presented in Figure 2. We used IM as basis for developing a strategy: to find a way in which to implement the guidelines for depression that suits the needs of the IPs.

### Step 1: Needs assessment

**Figure 3:**
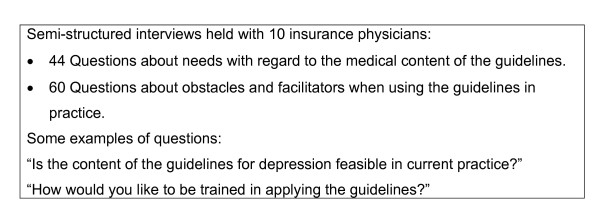
**Needs assessment**.

The key purpose of the *needs assessment *was to assess the needs of the IPs with regard to the guidelines for depression, as well as their opinions about the implementation of the guidelines at their place of work within the Institute. Interviews were held with 10 IPs working in practice (see Figure 3). They were asked to provide their opinions on: the content of the guidelines for depression, the possible obstacles, and the support, needed when using the guidelines in daily practice.

### Step 2: Program objectives

The program objectives were based upon the needs assessment mentioned in the first step. The expected outcome of the implementation strategy was defined. What should be changed in the behaviour of the program participants (IPs) and what should be changed in their environment (the Institute)? Learning objectives for the IP were related to the following personal determinants: knowledge and skills, attitude, self-efficacy, and expectations. Change objectives were related to the following environmental determinants: availability, uniformity, and support. We connected the program objectives, the learning objectives and the change objectives. This approach enabled us to define the concrete objectives, for which implementation could be developed. The main objective of the program was, to develop a strategy for the implementation of the guidelines for depression that suits the needs of the IPs

### Step 3: Selecting theory-based methods and practical strategies

**Figure 4:**
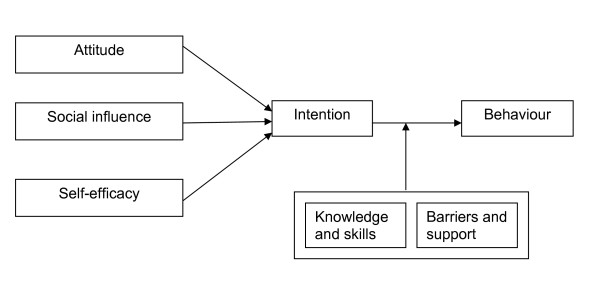
**The ASE model, as defined by De Vries **[24].

In this step, suitable theory based methods and practical strategies were sought, in such a way that the chosen implementation reflects the scientific literature and evidence. These methods were subsequently translated into practical intervention strategies, the effectiveness of which already had been scientifically demonstrated. Learning objectives were defined for each of the personal determinants, and change objectives for each of the environmental determinants. The learning objectives, as laid down in the personal determinants of the IPs, can be achieved if the IPs are willing to change their behaviour. The barriers or the support in the process of guidelines implementation at the Institute can influence the change objectives for the environment. For this reason we looked for a theoretical model that describes behaviour, and how the environment influences behaviour. We used the Attitude, Social Influence and Self-Efficacy (ASE) model, derived from the Theory of Planned Behaviour (TPB) [22,23]. The ASE model describes how a person's attitude, social influence and self-efficacy (i.e. personal effectiveness) influence behaviour, as is shown in Figure 4[24].

As used in this research setting, the ASE model may be explained as follows: the *behaviour *required from the IP consists of correct application of the guidelines for depression when assessing clients with depressive symptoms.

The *intention *to behave as described is determined by: 1) the IP's *attitude *to the use of guidelines in general, and the guidelines for depression in particular; 2) the *social influence *exerted by the physician's colleagues, and by the staff and managers who influence use of the guidelines, and who may influence availability and uniformity in using the guidelines; 3) the *self-efficacy *of the IP, or his/her confidence in his/her own ability to successfully apply the guidelines in practice [24]. The intention to use the guidelines does not necessarily result in their use in practice, i.e. the behaviour that is sought. The translation of intention into action is influenced by *barriers and support*, and by the existence of *knowledge and skills *(which can be increased by training) within the process and the organization. In the following steps we proceeded from theory to practical strategies; and from practical strategies to the intervention.

### Step 4: Program plan

The program plan for the implementation strategy was developed on the basis of the preceding steps: needs assessment, the matrix from step 2, and the theoretical model from step 3. Input for the program development process was obtained from semi-structured interviews and consultation rounds with 40 experts. These experts were mainly regular IPs, IPs responsible for appeal cases, (regional) staff physicians, and a few psychiatrists, training experts, and members of the management of the Institute. Preparing for these interviews and consultation rounds, we studied the disability assessment reports made by IPs to find out whether or not the main elements of the guidelines for depression could be found in the reports. Firstly, we had to look for the main elements of the guidelines for depression in the IP reports. Secondly, if we succeeded in finding them, these main elements could generate input for the training design. Finally, the main elements formed the basic assumption for the development of performance indicators (PI). In this investigation we screened IP reports on indicators of application of the guidelines for depression according to the saturation procedure. We screened reports until we reached the point, at which no new indicators for application of the guidelines could be found. Knowing that we could use the IP reports in our study, we designed a program plan, which incorporated the opinions of experts that we consulted. This program plan covered several aspects, such as PIs, instruments, training, and knowledge dissemination. In this stage we tried to match the implementation strategy with the needs and performance objectives of the IPs.

### Step 5: Planning the program implementation

After designing the program for the implementation strategy, we scanned the previous steps with a focus on objectives, methods and strategies, to ensure adoption by the IPs. A study of literature on the effects of implementation strategies was used to develop a suitable implementation strategy, consisting of instruments, training, testing and feedback [25]. Given the context of the Institute, will the implementation strategy receive broad support, or could there be any obstacles in the implementation process? We consulted both users and stakeholders at the Institute regarding the content of the program and the implementation strategy. The users we consulted were the same 10 IPs who participated in the needs assessment. We then consulted six stakeholders at the Institute, i.e. the medical adviser, two regional staff physicians and three regional managers.

### Step 6: Evaluation

The intervention map can be used as an evaluation model for the development of the process, and for the effect of the corresponding intervention. In a future study we will evaluate the efficacy of a specific training in the implementation of the guidelines for depression in a two- armed RCT. The primary outcomes of the RCT will be the quality of the IP reports of the assessment of a claimant with depression, and the adherence of the IPs to the guidelines for depression. The outcomes of this RCT will be measured with performance indicators (PI) and questionnaires.

## Results

### Step 1: Needs assessment

Semi-structured interviews were held with 10 IPs. Almost all of these 10 IPs considered the guidelines to be useful as a reference, but indicated that they lacked information that is needed for direct use in practice. The specific items, representing the most important needs mentioned by the IPs with regard to the guidelines, are summarised in Table 1.

**Table 1:** The needs of the insurance physicians with regard to guidelines for depression

Diagnostics	A list of the DSM IV criteria for depression and the DSM IV criteria for the most relevant differential diagnostic psychiatric disorders
Psychiatric examination	A list of psychiatric examination items on a desk mat

Seriousness depression	A method with which to determine the seriousness of depression in a uniform way. The Hamilton Rating Scale of Depression? (HRSD)

Seriousness and disability	Expert opinion to clarify the relationship between the seriousness of the disorder and the assessed disability

Prognosis	Need for evidence-based information about periods of recovery from depression in relation to treatment and co-morbidity

Guidelines for depression and other standards	Expert opinion on the relationship between the guidelines for depression and the standards: "Full disability entitlement on medical grounds" and "reduction in working hours" for partly disabled claimants

Coping styles	Information about personal characteristics and coping styles and how to distinguish between disease and behaviour

The IPs' wishes regarding implementation of the guidelines for depression were also established. They needed expert education. This should preferably be interactive training provided by experts, paying attention to practical relevance. The IPs wanted instructions on how to use the instruments, such as a desk mat listing all diagnostic criteria, and psychiatric questionnaires based on case histories. In conclusion, the IPs wanted to be trained in applying the guidelines in practice with the help of experts and practical instruments. The IPs' wishes regarding the training module to support the guidelines are summarized in Table 2.

**Table 2:** Implementation strategy: insurance physicians' wishes regarding educational training and support in the use of the guidelines

Training module	Form, implementation	Method
Introduction to the guidelines for depression	Experts from the curative sector and insurance physicians with knowledge of depression	Presentation of problems from curative and insurance-medical viewpoints; mutual questioning regarding experience and vision

Materials, tools	Summary card listing all diagnostic criteria	Practise in the use of the materials, and case histories

Case-histories	Group discussion and practise in applying the guidelines	What constitutes a good assessment? What is unclear? Why?

Work ability assessment	Insurance physician and psychiatrist/psychologist-researcher	Scientific insights, experiences, focus on problems (LFA)

Information on treatment possibilities	Experts from the curative sector	Current thinking on appropriate treatment. What questions can the insurance physician put to the curative physician?

Carrying out and interpreting psychiatric tests	Psychologist, psychiatrist	Different presentation in ethnic minorities (a high proportion of the claimants)

Detailed explanation and interpretation of the HRSD questionnaire	Psychologist, psychiatrist	Practise in the use of the questionnaire

Feedback	Insurance physician and guidelines author/researcher	Feedback from the profession; opportunity to ask questions

LFA = List of Functional Abilities; HRSD = Hamilton Rating Scale of Depression

### Step 2: Program objectives

In this step we defined the behavioural and environmental determinants of he program, and translated them into performance objectives for the IPs and change objectives for the Institute. The IPs should learn how to use the guidelines for depression, and they should consider themselves capable of applying the guidelines in practice. By using and applying the guideline the IPs should believe that they could improve their performance with regard to their work ability assessments of claimants with depression. The Institute should increase the availability of the guidelines for the IPs, and should support the implementation by putting more emphasis on quality instead of productivity. Staff physicians should generate a strong influence in the use of the guidelines, by monitoring the IPs' reports on guideline adherence. The expected behaviour of the IPs is, that they will learn to apply the guidelines for depression. All the determinants of this behaviour were presented in a matrix (Table 3), crossed with the program objectives, showing the specifications of the program objectives for the IPs.

**Table 3:** Program objectives, learning objectives and change objectives

Program objectives for insurance physician	Learning objectives for insurance physician (IP)'s personal determinants				Change objectives for environmental determinants	
	
	Knowledge and skills	Attitude	Self-efficacy	Expectations	Availability and uniformity	Support
IP makes thorough investigation and records findings transparently in the report.	IP has sufficient knowledge and skills to understand the guidelines and to implement it in practice.	IP accepts the guideline as a practical resource and a useful source of information.	IP considers him/herself capable of applying the guidelines in practice.	IP believes that use of the guidelines can make his/her examinations more thorough and transparent.	IP is trained to use the guideline and has the opportunity to practise using it during the training and subsequently in practice.	IP's quality-oriented activities are supported by National Institute for Employee Benefits Schemes by putting the emphasis on quality instead of productivity. Access to evidence-based medical info via Internet and/or library.
To do so, IP uses the guidelines in order to ensure quality and uniformity of the assessment.	IP has the skills to perform the examination in line with applicable requirements.	IP supports the profession's general objective of fair assessment based on thoroughness, quality and uniformity.	IP consider him/herself capable of investigating issues associated with the assessment and obtaining guidance from the guideline, literature or colleagues	IP believes that the quality and uniformity of his/her work ability assessments will be enhanced by the information in the guidelines.	Case histories are discussed amongst colleagues by reference to the guideline, enabling IP's to ask questions and learn from one another.	Staff of the Institute support IP in use of the guidelines and related activities. Staff physician encourages use of the guidelines, by testing the IP's reports on guideline adherence.
IP uses evidence-based information to support work ability assessment	IP has sufficient evidence-based knowledge to recognize and address any lack of skills.	IP sees the guideline as a means to realizing the objective.		IP believes that the information in the guidelines will help him/her make more evidence-based work ability assessments.	Staff physicians provide all IP's with performance feedback and work with IP's to define individual learning programmes so that all attain a similar level.	Netherlands Association of Insurance Medicine supports IP's quality-oriented activities and encourages use of the guidelines.

IP = insurance physician

### Step 3: Theory-based models and practical strategies

Practical interventions were chosen to realize the learning and change objectives mentioned in the Matrix 1. Subsequently, by putting the personal and environmental determinants in another matrix with the learning objectives, theory-based methods, and practical strategies, the required conditions for the development of the intervention were obtained. These methods and strategies were incorporated in the development of an interactive training with feedback. Adequate feedback on the performances of the IPs in the training should confirm their expectations that i.e. using the guidelines will contribute to more evidence-based assessments. IPs first must be aware of the guidelines, then become familiar with the guidelines, and finally believe that they are capable of working with the guidelines. IPs should be facilitated and stimulated by their environment in applying the guidelines, offering them training that suits to their needs. The Institute and the Netherlands Association for Insurance Medicine should support and involve the IPs in the development and implementation of guidelines. Determinants of learning and change objectives, and the associated strategies matched with theory-based methods are presented in Table 4.

**Table 4:** Determinants of learning and change objectives and the associated strategies

Determinant	Learning objectives for the insurance physician	Theory-based method	Practical strategy
Knowledge	Familiarity with the content of the guideline	Dissemination of training material Active learning from experts	Making guideline available in combination with practical instruments

Skills	The ability to apply knowledge in practice	Interactive group training	Interactive training in use of the guidelines

Attitude	Willingness to accept the guidelines and use them to improve quality	Persuasion by opinion leaders	Benefits highlighted during training and by staff and the Netherlands Association for Insurance Medicine

Self-efficacy	Belief in ability to use the guidelines in practice and finding answers to questions	Performance-related feedback	Positive individualised feedback during training and subsequently in practice, assistance with questions

Expectations	Expectation that the guideline will contribute to more evidence-based assessments	Individualized feedback and group performance audit data	Training in use of the guidelines with exercise case-histories, feedback at group and individual level

	**Change objectives for the environment**		

Availability	The ability to practise, ask questions and work on personal performance	Feedback, personal improvement, planning	Practice in training, feedback on performance, support with questions

Uniformity	All insurance physicians covered by similar requirements	Quality-monitoring and quality-management	Staff physician appraises all insurance physicians using the same indicators

Support	Support from colleagues, staff, management and professional association, facilitation and, where necessary, amendment of the work process	In-built process reminders, quality management, support from opinion leaders	Quality evaluation by management, staff quality-oriented direction, promotion by the Netherlands Association for Insurance Medicine

### Step 4: Program plan

Research on the reports made by IPs when they assessed a claimant with depression, showed that these reports, indeed, did include the main elements of the guidelines for depression. Saturation was achieved after 30 reports. Even without training IPs in the use of the guidelines, elements of the guidelines appeared in the IPs' reports. That made it possible to develop PIs for testing the reports for elements of the guidelines in the baseline situation. After this saturation procedure, we knew that we could use the IPs' reports to evaluate their implementation of the guidelines for depression. In addition, having found the main elements of the guidelines in the IPs' reports, we could determine the starting point for the design of the training. The planning of the implementation strategy was prepared and involved the following steps (see a, b and c below).

#### a) Development of prototype instruments

The results of the interviews with the IPs were used in the development of the prototype instruments. With a view to aligning the instruments with the objectives of the guidelines, we consulted the adviser and secretary of the Health Council's Subcommittee on Depression. To supplement the guidelines for depression, a study was made of the literature on co-morbidity, prognostic risk factors, and the work capacity of individuals with depression. The result was a toolbox: a collection of instruments intended to facilitate application of the guidelines (see Table 5).

**Table 5:** Content of the toolbox

Desk mat	Diagnosis and differential diagnosis based on the DSM-IV Assessment of the severity of depression Psychiatric examination Psychiatric co-morbidity Somatic co-morbidity Effective treatment methods Risk factors in relation to the severity and duration of disabilities The International Classification of Functioning, Disability and Health model (ICF model) [26] Key findings of the literature study referred to above
Checklist	Items referring to the main points of the guidelines for depression

HRSD [27]	Assessing the severity of depression

HRSD = Hamilton Rating Scale of Depression

The *desk mat *showed on the front summarised information on the most essential points of the guidelines for depression. The back of the desk mat showed the relationship between the various relevant risk factors in a diagram based on the International Classification of Functioning, Disability and Health model (ICF model) [26], which was also used in the development of the insurance medicine guidelines. The ICF model is the framework within which the insurance physician operates when assessing the work ability of a disabled employee. Furthermore, a *checklist *contained items referring to the main points of the guidelines, such as the DSM IV criteria, seriousness of the depression, co-morbidity and treatment. When assessing a claimant with depression, the IPs can check all the relevant items and to make sure that they have not forgotten anything. Finally, the *Hamilton Rating Scale for Depression *(HRSD) [27] was added to the toolbox to assist the IP in the assessment of the severity of the depression at the time of the examination. Use of the HRSD needs to be included in the training for IPs in connection with implementation of the guidelines.

#### b) Refinement of the prototype instruments with help of a group of IP users

The toolbox was shown to a group of users, consisting of 10 IPs. A questionnaire was used to establish their opinions with regard to the practicability, quality, content validity and added value of the instruments, as well as how easy they were to understand and the extent to which they allowed room for professional assessment. On the basis of the feedback from the user group, we searched for additional literature and adapted the instruments where necessary. This resulted in an amended and compressed desk mat and a check list. Also added to the toolbox was a consensus-based *list of the main abilities *that were thought to be associated with the work ability of employees with a major depressive disorder, and that could also be associated with the items of the HRSD [28].

#### c) Development of the training

A separate group of experts, which included psychiatrists and training experts, was set up for consultations regarding the design of the training. This round of consultations resulted in the final training design as follows.

The IP should be given practical instructions about application of the guidelines for depression. This should include instructions on how to arrive at an evidence-based assessment of a depressive claimant's functional abilities, based on the knowledge presented in the guidelines. The learning objectives of the training appeared to be that the participating IPs trained their skills in making a diagnosis of depression, how to assess the severity of the depression and the disabilities, and how to report on the relationship between these issues. Meanwhile, they should learn how to give their assessment reports a solid base. To this end, the IPs should be provided with the aforementioned instruments. The training should start with a knowledge test based on the guidelines for depression. A psychiatrist who is familiar with the insurance-medical assessment system should then explain a number of important aspects of the assessment of depression on the basis of an interesting and recent case concerning an immigrant employee with an atypical presentation of depressive symptoms. Focus points should include diagnostics, the distinction between behaviour and disease, symptomatology, the relationships between symptoms and disabilities, assessment of the severity of the depression (including use of the HDRS), treatment, progression of the condition, and co-morbidity.

**Figure 5:**
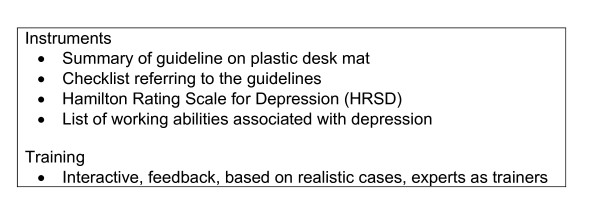
**Program plan**.

Subsequently, with a video recording of case study, an IP trainer should describe the practical aspects of using the instruments. The group of participants in the training should then be divided into subgroups, each focusing on a different part of the guidelines, to make assessments of the presented case. The relationship between the existing medical standards, "full disability entitlement on medical grounds", "reduction in working hours", and the guidelines for depression should be explained by the trainer. Different coping styles and personal characteristics of claimants should be integrated in the realistic cases presented during the training. In the training, the IPs should learn how to differentiate between the various types of coping styles of claimants with depression. Interactivity between the sub-groups and self-activation should alternate frequently, while feedback should be given by the trainer in a attempt to achieve the learning objectives for all the participants. When writing down their findings and conclusions, the participants should be instructed to use the essential elements of reasoning. Finally, the training day should end with an evaluation. In this kind of training design, the number of participants for each group should be limited to 20, because it is characterized by intensive communication, with feedback and interactivity between the participants and the trainer. The program plan is summarized in Figure 5.

### Step 5: Program implementation

We were interested in the opinions of experts, groups of users, and management and staff about implementation of the guidelines at the Institute, so that we could build up a picture of the context within which the IP works. The management and staff stated that, by implementing guidelines, they meet the requirements of the Ministry of Social Affairs. Furthermore, by implementing guidelines, the Institute might obtain more public support, and might face fewer complaints and appeals from claimants. Nevertheless, implementing guidelines could induce a loss of production. The IPs were pleased with the fact that, by carrying out research on the implementation of insurance medicine guidelines, attention will be paid to the quality and the content of their work. On the other hand, they realized that adopting guidelines might be a complex process for them, because they had to integrate working with the guidelines in their daily routine. The IPs had no previous history of working with guidelines. The IPs were in particular asked, to identify obstacles to and support for the use of guidelines for depression, and how the obstacles might be removed. One commonly identified obstacle to the use of a guideline was the emphasis placed on the quantity of the number of disability assessments to be made by an IP, which was imposed by the Institute. It was suggested that the Institute could facilitate the use of guidelines by placing more emphasis on quality, rather than quantity. Applying the guidelines thoroughly takes time, and productivity requirements limit the time that is available. The Institute was regarded as a productivity-driven organization. It was stated that staff physicians could stimulate the IPs to use the guidelines by giving them clear instructions about how to use them.

From the literature [29] and from consultations of decision-makers and implementers, it was found that the PIs for the guidelines can support the staff physicians in checking the IPs' reports on their adherence to the guideline. That would be a strong facilitator for using the guidelines according to the interviewed physicians. Furthermore the PIs could be used for feedback after training the IPs in the use of the guidelines, which was one of the needs of the IPs. By implementing guidelines, the decision-makers meet the requirements of the organization and the Ministry, but they might be faced with a loss of IP productivity. The IPs put more emphasis on quality by the implementation of guidelines, but wondered if they were capable enough of using the guidelines. The positive and negative features of the program implementation, as identified by the decision-makers, implementers and IPs, are summarized in Table 6.

**Table 6:** Positive and negative features of the program implementation for various parties concerned

Parties involved	Positive features of program implementation	Negative features
**Decision-makers**		
Management of Socio-Medical Department	More public support Meets ministry requirements Fewer appeals and complaints	(Initial) loss of production Research takes time

**Implementers**		
(Regional) managers	Increased quality Fewer complaints	Loss of production, possibly temporary Appeals are not reduced
(Regional) staff physicians	Better-quality assessments More transparent decisions Easier test procedure to check reports	Guidelines must not be rigid Legal status of guidelines: implication for appeals?

**Users**		
Insurance physicians	Useful guidelines and EBM information Guidelines with instruments tailored to IPs in practice Focus on quality and content Scope for professional assessment maintained	Learning a new approach takes time; integration in personal routine is an effort Stricter requirements made regarding examination and reporting Will the extra workload be appraised and supported by staff and management? Legal status of guidelines: implication for appeals?

**Concerned**		
Claimants	More thorough and uniform claim assessment	Longer, more structured consultations (not necessarily a drawback)

**Researchers**		
Experts	Influence on content	Time input

### Step 6: Evaluation plan

**Figure 6:**
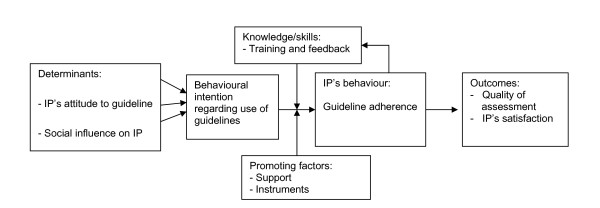
**Research model, schematically represented on the basis of the ASE model**. IP = Insurance Physician

The efficacy of the strategy for implementation of the guidelines, described in this article, will be compared to traditional implementation in a two-armed RCT. One group of IPs will receive specific training in applying the guidelines for depression, while the other group will continue with the traditional implementation of the guidelines. Hence, the specific training for the IPs will be the intervention in this RCT. Outcomes will be measured by PIs and questionnaires. The PIs measure the primary outcome, i.e. the behaviour of the IPs with regard to the guidelines, defined as: the quality of the IPs' reports of the assessment of a claimant with depression. The questionnaires not only measure the IPs' adherence to the guidelines, but also their satisfaction with the guidelines, which is a secondary outcome of this study. The questionnaires were developed on the basis of the literature and the ASE model [30,31]. In the RCT the PIs and the questionnaires will determine the performance objectives of the IPs before and after the intervention. The process of the RCT and the specific training of the IPs in applying the guidelines will be evaluated in a process evaluation. The data for the process evaluation will be collected with by means of specifically developed evaluation questionnaires. In order to illustrate how the effects of the implementation will be measured, the research model is presented in Figure 6.

## Discussion

The aim of this study was to develop an implementation strategy to improve guideline adherence and the quality of the assessments made by IPs of the work ability of employees with depression. IM has its origin in public health, and in particular in health promotion programs. More recently IM has found its way into the field of occupational health medicine, where it has been used for Return to Work (RTW) interventions. The results of this study show that IM proved to be useful in the development of a strategy for the implementation of the insurance medicine guidelines for depression.

### Strengths and weaknesses

#### Strengths

IM provides an implementation strategy framework in which a solid theoretical base and the participation of the IPs is integrated. The IPs will be more motivated to adopt the guidelines for depression if they are good compatible with daily practice and suit to their needs. By following all steps in the IM process, and with the help of 40 experts in the development of the instruments, performance indicators and training, we tried to achieve the practical feasibility of the guidelines for the IPs. We involved not only IPs and experts in the IM process, but also staff physicians, regional managers, the medical adviser and the top management of the Institute.

#### Weaknesses

Generalization of the outcomes from IM studies might be difficult, because the IM process takes the local context into account. In our study, however the local context is set by the Institute: a national organization in which IPs assess the work abilities of claimants. Therefore, the outcomes of our study can only be generalized to other countries in which there is a central organization for employee benefits and IPs working with guidelines. Another weakness is that claimants with depression were not represented in this study. Nevertheless, we think, that a justifiable, careful and transparent assessment of work ability, in accordance with the guidelines, might be more acceptable for the claimant, than assessments without guidelines.

### Comparison with other studies

IM studies in insurance medicine are scarce, and only one has been published [20]. In that study, IM was used for the development of an RTW intervention program, whereas we used it for the development of a strategy for the implementation of the guidelines for depression by IPs. IM has been used as a systemic approach in designing a quality improvement intervention for general practitioners (GPs) [32]. In that study, using IM in the process of implementing guidelines for GPs, although time-consuming, appeared to be worthwhile. In Belgium, research has been carried out on the application of EBM and guidelines among IPs [33]. In that study, the IPs' knowledge about EBM and practical guidelines was found to be rather poor. Therefore, the authors recommended that high quality EBM and practical guidelines should be structured in such a way that they are useful for IPs. In our study we tried to achieve that aim with the added value of using IM. We tried to meet to the needs of the IPs, and we integrated EBM in the development of the instruments. This approach resulted in a tailor-made intervention: educational training for IPs in applying the guidelines for depression. However, with or without training, the application of guidelines by physicians remains a complex process, lacking in-depth knowledge about which factors are decisive in that process [13]. Integrated in the third step of the IM process, the ASE model, derived from the Theory of Planned Behaviour (TPB) [22,24] appeared to be suitable to cover those factors. The adherence of physicians to the guidelines has been related to TPB in several studies [10,34-36], and the overall conclusion was that health behaviour theory can be useful for improving adherence to clinical practice guidelines. Cabana [34] reviewed 76 studies on barriers to guideline adherence among physicians. From his review he compiled a list of barriers in physician adherence to guidelines. In our study we tried to overcome barriers in the adherence of physicians to guidelines by using IM for the development of our implementation strategy. The IPs will be made familiar with the guidelines for depression by a specific training. The guidelines were made more accessible for the IPs with the help of practical instruments. Bearing in mind the recommendations made by Grol in a review [37], we provided the IPs who participated in our study with a well-designed and well-prepared program for implementing the guidelines. In another review focussing on physicians' attitudes to guidelines [38], the authors stated that high satisfaction with guidelines does not necessarily results in practice changes. Individual physicians would not make significant changes without the necessary educational, organizational and structural changes in the health care system [38]. By using IM we tried to encourage the IPs to use the guidelines, taking into account all the aspects of behavioural change mentioned above.

### Practical relevance

The IM method cannot only be applied for implementing the guidelines for depression, but also for other insurance medicine guidelines at the Institute, and for guidelines in other disciplines outside the Institute. We expect that by using IM to develop a strategy for the implementation of insurance medicine guidelines, adherence of the IPs to the guidelines will improve. The educational training, as developed for the guidelines for depression, could be adjusted and prepared for the implementation of other insurance medicine guidelines. The implementation of the guidelines and the development of the PIs, has made quality testing possible. Auditing professional quality is a challenging issue and the social and professional need to measure quality has increased considerably in recent years. Occupational health processes have long been audited by means of indicators [10,39,40], and now indicators will be introduced into the field of insurance medicine to monitor IPs' assessments of the work ability of claimants. Transparency of professional decision-making can provide a basis for quality improvement and our study design is consistent with this trend of auditing quality improvement.

Further research is recommended to determine, whether an IM based strategy for the implementation of insurance medicine guidelines actually contributes to IP adherence to guidelines. We expect that the results of the RCT and the process evaluation will provide us with an answer to that question.

## Conclusions

This article describes the use of IM in the development of a strategy for the implementation of the insurance medicine guidelines for depression.

Although the implementation strategy we developed has yet to be evaluated, we may already conclude that the use of IM made it possible to develop guideline support instruments that are tailored to insurance medical practice. The instruments and PIs that were developed meet the needs of IPs, and take into account the context in which they will be used.

## Competing interests

The authors declare that they have no competing interests.

## Authors' contributions

FZ wrote the manuscript. AJMS, KCR and JRA contributed to the manuscript. AJMS and JRA designed the study. AJvdB commented on the manuscript and will act as guarantor of this study. All authors have read and approved the final version of the manuscript.

## Pre-publication history

The pre-publication history for this paper can be accessed here:

http://www.biomedcentral.com/1471-2458/11/9/prepub
